# Lessons learned about development and assessment of feasibility of tools for health and rehabilitation services

**DOI:** 10.1186/s40814-023-01424-w

**Published:** 2024-02-02

**Authors:** Harsha Kathard, Rizwana Mallick, Tracey-lee Cloete, Anthea Hansen, Lehana Thabane

**Affiliations:** 1 Faculty of Health Sciences, University of Cape Town, F45 Old Main Building, Groote Schuur Hospital, Observatory, 7925 Cape Town South Africa; 2 Department of Paediatrics and Child Health, Faculty of Health Sciences, Children’s Institute, University of Cape Town, 46 Sawkins Road, Rosebank, Cape Town, 7700 Cape Town South Africa; 3 Centre for Health Professions Education, Stellenbosch University, Matieland, 7602 Stellenbosch South Africa; 4 Faculty Health Sciences, Mc Master University, 175 Longwood Road South, Hamilton, ON L8P 0A1 Canada

**Keywords:** Pilot, Feasibility, Tools, Contextually relevant, Equitable service delivery

## Abstract

**Background:**

Given the dire need for health and rehabilitation services internationally, exacerbated during the COVID-19 pandemic, there is a critical need to develop tools to support service delivery. This need is palpable in the Global South where tools developed in Eurocentric contexts are not always adaptable, applicable, or relevant. It is for this reason that the researchers present three case studies of tool development using pilot and feasibility studies in South Africa and share the lessons learned from these studies.

**Objectives:**

To describe three case studies that developed new tools for health and rehabilitation services using pilot and feasibility studies.

To synthesize lessons learned from these case studies on the development of tools.

**Method:**

The researchers describe three case studies that were developed. The case studies are summarized as follows: aims and objectives, context, problem, study design, findings, and what happened after the study. Thereafter, a qualitative cross-case analysis was conducted by the researchers to generate themes.

**Findings:**

The case studies are described individually and followed by themes identified through cross-case analysis.

**Discussion:**

The lessons learned are discussed. It is essential to develop new tools and protocols, motivated by the need for equitable and contextually relevant practices. Partnerships and collaboration with end-users are critical for success. A critical, scientific process is essential in developing new tools. Pilot and feasibility studies are invaluable in developing tools and assessing the feasibility of tools and implementation. The goal is to develop practical, usable tools and protocols.

**Conclusion:**

Through the lessons learned, the researchers are hopeful that the international health and rehabilitation professions will continue to strengthen the scientific development of contextually relevant tools and resources.

## Background

There is a need to provide health care in a manner that is responsive to the needs of society, which requires a reimagination of the tools used in health care. Health and rehabilitation professions in South Africa are actively developing tools that are relevant to its context to serve marginalized communities more equitably [1–4]. South Africa has a two-tiered public–private health system mired in deeply entrenched structural inequalities, [5, 6] and as a consequence, rehabilitation service delivery is also unequal [7]. Due to the ever-increasing burden of disease, now further exacerbated by the COVID-19 pandemic and poor living conditions, the burden of disability is increasing [8]. It is estimated that one in three people internationally will need rehabilitation services during their illness or injuries [9], and therefore, comprehensive primary health care services must include rehabilitation as a priority, particularly at a community level [10, 11].

The tools (a broad term researchers use in this paper to describe protocols, interventions, and instruments), currently available in South Africa, were mainly developed for application in Western cultural contexts. However, when these tools are applied inappropriately to communities they were not designed for, there are significant negative consequences [4, 12–14]. The use of appropriate tools is a matter of social justice. Given this exploratory context with several unknowns, the researchers reflect on lessons learned in three case studies using pilot and feasibility studies for the scientific development of tools in a context of many uncertainties [15]. This commentary seeks to make a contribution to the literature related to the use of pilot and feasibility studies to develop tools that are crucial in advancing equitable rehabilitation service delivery.

The philosophical approach taken across the case studies resonates with the Re-Aim framework [16] which encourages scientists to be explicit about context and strategy, include qualitative research methods, and support the development of user-friendly and human-centered approaches.

## Objectives

The objectives of this paper are to:

1. Describe three case studies in which researchers aimed to develop contextually relevant tools
2. Discuss the lessons learned about the scientific process of developing tools using pilot and feasibility studies

## Methods

### How the case studies came about

The case studies were developed through researchers’ identification of key service delivery challenges as they worked as university clinical educators in community settings to strengthen rehabilitation service delivery.

#### Case study 1: developing and testing a hearing screening protocol [17]

The researcher was concerned about the lack of contextualized hearing screening guidelines (at the time of the study) and how this contributed to inadequate school-based hearing screening services in the region [18]. The state of school hearing screening practice was especially concerning when considering the adverse effects of hearing impairment on the communication and learning ability of school-aged children [19]. Through collaborative work with the school health team in the district, and nurses in particular, key role players identified an urgent need for a contextually relevant hearing screening protocol which led to this case study.

#### Case study 2: pilot study of Classroom Communication Resource (CCR) [20]

The need for school-based intervention for children who stutter was identified by student clinicians and teachers in a marginalized community where speech-language therapy services were severely limited. Having identified this need, the students designed an intervention (Classroom Communication Resource) which was refined through further [21–26] studies. Thereafter, this case study was conducted to inform the feasibility study for an RCT.

#### Case study 3: the development of a rehabilitation and health information tool (RHIT) [27]

This case study was developed in response to the need for a user-friendly, contextually relevant tool to gather health and rehabilitation information from persons with disabilities. The need was identified by community rehabilitation workers providing continuity of services to persons with disabilities who required community-based care. They required the tool to gather information on the rehabilitation and health needs of persons with disabilities, plan interventions, and monitor progress. Hence, the study was conducted in partnership with community rehabilitation workers who were being trained as part of a pilot program at the University of Cape Town, Department of Health and Rehabilitation, aimed at strengthening community-based rehabilitation support.

### Summarizing case studies

Each case study was summarized from published papers (case studies 1 and 2) and from the completed Masters’ studies (case study 3) The summary of each study included the aims and objectives, description of context, problem identified, study design, and findings. In addition, the researchers describe what happened since the conclusion of the studies.

### Cross-case analysis

The researchers analyzed the summary of each study and then conducted an interpretive cross-case analysis to generate themes emerging across cases. The discussion focuses on lessons learned from these themes.

### Findings

#### ***Case study 1***

See Table 1.

**Table 1:** Developing and testing a hearing screening protocol

Aims/objectives	1. To develop a hearing screening protocol for grade 1 learners in schools from the local school district 2. To determine the feasibility of applying the proposed protocol in the school context
Context (at time of study)	The study was conducted in Western Cape Province in South Africa where there are vast economic disparities evident in a schooling system where over 80% of schools are categorized as public entities serving the majority of the population. Over 40% of these public schools have limited financial and human resources and experience difficulties accessing health care, including school-based hearing health services
Problem	The nurses, as key role players in school health, identified the need for an effective hearing screening protocol which was sensitive to the limited time and human resources constraints
Study design (include sample size)	The mixed methods study was designed in two sequential phases, namely the development phase and feasibility testing Phase 1: Development phase Methods: A *focus group discussion* was conducted with 5 nurses to identify their needs and test properties suited to their context A *systematic literature review* was conducted to appraise published evidence of effective hearing screening tests and identify two potential hearing screening tests to meet the nurses’ contextual needs *Expert panel* comprising 2 school nurses and 2 pediatric audiologists reviewed the synthesized data from the focus group discussion and systematic review to identify a screening protocol suited to the context. They used a Likert-type scale for ratings Phase 2: Feasibility of implementation After nurses were trained on how to implement the hearing screening protocol the feasibility was assessed through: - *Observations* of 4 nurses implementing the protocol with 100 grade 1 children in the school context, using an observation schedule - Conducting a *test-re-test reliability and inter-tester reliability* of the hearing screening using a sample of 45 randomly selected children - Determining *the sensitivity and specificity* of the test by comparing the test results generated by the nurses with gold standard diagnostic audiology findings
Findings	Phase 1: Development phase The required test properties identified were as follows: (1) quick to administer, (2) easy to administer, (3) easy to interpret, (4) resistant to background noise, and (5) yield accurate results The literature review identified the distortion-product oto-acoustic emissions (DP-OAEs) and pure tone testing as suitable tests The expert panel proposed the DP-OAE as the test most likely to succeed in the context Phase 2: Feasibility of implementation The protocol was found to be feasible for nurses to implement The inter-rater reliability between nurses was generally high and consistent with international findings The sensitivity of DP OAE testing was 57% (warranting further exploration) while the specificity was 97%
What has happened since the conclusion of the study	Upon study completion, the findings were presented to the Western Cape School Health Forum and the proposed protocol was well-received. While the challenges of implementing the protocol within the current school health program still remain, the collaborative research process has increased the school nurses’ willingness to utilize the proposed protocol

#### Case study 2

See Table 2.

**Table 2:** Pilot study of Classroom Communication Resource (CCR)

Aims/objectives	To determine: 1. Recruitment rates of schools and participants, and the dropout rate of participants 2. Treatment effect of attitudes towards stuttering among grade 7 learners based on the Stuttering Resource Outcome Measure (SROM)
Context	Schools in higher and lower quintiles in the Western Cape metropolitan area participated in the study where the language of teaching and learning was English
Problem	The CCR intervention had been developed and refined to make it contextually relevant in the following ways: • It was shortened to one lesson because teachers did not have time to administer a multi-lesson program over several weeks. However, teachers were willing to continue discussions as and when the need arose • It could be administered in parts, e.g., story, roleplay, and discussion pieces to make it manageable for teachers and learners to engage with. Through exploration of learners’ experiences of stuttering as well as cognitive debriefing with learners, the CCR was modified to use concepts sensitive to the realities of learners. The cognitive debriefing sessions examined overall understanding and use of vocabulary and terms to improve the questionnaire. For example, “play time” was changed to “break time”. These modifications allowed the questionnaire to become more culturally and linguistically appropriate • It was adapted to be linguistically appropriate, i.e., children in South Africa who were learning English as an additional language • It encouraged a generative conversation between learners and teachers from diverse cultural contexts to participate and express their different worldviews that extended beyond stuttering around inclusion, acceptance, and diversity However, the researchers did not know its potential treatment effect over a longitudinal period and how feasible it would be to recruit and retain participants for an RCT
Study design (include sample size)	The pilot study used a stratified cluster randomized design The sample size was 401 with 149 learners included in the CCR intervention and 252 learners in the usual practice (control group) For the treatment effect, the schools were randomized with teachers using the CCR or usual practice. The stuttering resource outcome measure (SROM) was used at baseline, 1 month, and 6 months post-intervention The recruitment took place at the school level and then at the individual level. The dropout rate was noted at baseline and at 1 and 6 months post-intervention
Findings	The recruitment rate for school recruitment was 82% as 9 of the 11 schools invited participated in the study. While 601 learners were eligible and invited to participate in the study, 449 were recruited with 183 randomized to the treatment group and 266 to the control group. The drop-out rates for the learners at baseline were 23% (*n* = 34) in the intervention group and 6% (*n* = 15) in the control group At 1 month post-intervention and 6 months post-intervention, the drop-out rates declined further as follows: Intervention group: 7% (*n* = 10) at 1 month and 7% (*n* = 10) 6 months post-intervention Control group: 6% (*n* = 15) at 1 month and 17% (*n* = 44) 6 months post-intervention This outcome indicated that there were several procedural refinements required to improve the retention rate. These improvements included improved communication for consent, reduction in frequency of testing, and changing the test schedule to align with school activities Treatment effect estimate: the study found that the treatment effect was evident at the 6-month interval rather than the 1-month interval
What happened since the conclusion of the study	A randomized control trial was conducted as well as a further qualitative study with teachers to explore their experiences of the intervention. The RCT showed no statistically significant results at 6 months post-intervention with valuable input from teachers regarding how best to integrate the intervention within the curriculum. The CCR is available online for use by therapists and teachers

#### Case study 3

See Table 3.

**Table 3:** The development of a rehabilitation and health information tool (RHIT) [27]

Aims/objectives	AIM: to develop a contextually relevant resource tool to support the community rehabilitation workers in understanding and documenting how the rehabilitation and related health needs of persons with disabilities are met in home- and community-based settings Objectives: (i) to develop the content and domains of the rehabilitation and health information tool; (ii) to establish the validity (face and content) of the rehabilitation and health information tool; and (iii) to test the application of the rehabilitation and health information tool on a sample of persons with disabilities
Context	The study was conducted in the Mitchells Plain/Klipfontein area, Western Cape, as the community rehabilitation workers were part of the Western Cape Department of Health pilot training project deployed in this substructure. It is a low-income community, with high rates of unemployment linked to low levels of education. It has a high crime rate and alcohol, drug abuse, and gang-related activities are high (City of Cape Town, 2006)
Problem	The local policies in South Africa such as the National Health Insurance [28] and the Framework and Strategy for Disability and Rehabilitation of the South African Department of Health [29] emphasize the importance of mid-level health workers such as community rehabilitation workers as a key component in primary health care. However, this cadre of worker faces many barriers to providing effective health care, including the lack of resources and supervision structures to support their role in community-based rehabilitation. This study was conducted to develop a rehabilitation and health information tool (RHIT) with community rehabilitation workers
Study design (include sample size)	The study adopted a sequential mixed methods design. Phase 1: conceptualizing the RHIT Sample size: 6 participants in the focus group discussion (2 persons with disabilities, 2 expert health and rehabilitation practitioners, 2 community rehabilitation workers Methods: document review and focus group discussion with expert panel Phase 2: Pilot test of the applicability of RHIT Sample size: 54 participants (adult persons with a disability requiring home care) Methods: the RHIT was administered face-to-face by 10 trained rehabilitation care workers who provided feedback on the application of the tool
Findings	Phase 1 identified 12 content domains for the RHIT: overall health, self-care, mobility, communication, relationships, sexual health, general tasks, access to health information, health behaviors, health safety and security, spirituality, and others Presented through 9 close-ended questions and 4 sub-questions Following the focus group with the expert panel, the RHIT was revised further to refine the socio-demographic questions such as living arrangements, type of housing, and living environment; additional questions were added to rehabilitation and health needs, and the wording of the questions was simplified to improve linguistic accessibility for community rehabilitation workers. These processes enhanced the face and content validity of RHIT Phase 2: Field testing of the RHIT indicated that while it was contextually relevant, it covered the content areas and importantly highlighted rehabilitation needs. It was useful for the community rehabilitation workers as it did not require scoring. It assisted in generating a conversation on health needs, intervention planning, and monitoring over time. However, it needed to be shortened and there was a need for further training of workers on the administration of some content areas, e.g., sexuality, as well as on recording information
What happened after the study	The RHIT requires further refinement, follow-up testing, and validation before it can be formally adopted as part of the rehabilitation care worker’s practice. This process has not yet commenced. However, the lessons learned from this study have been incorporated into the training of these workers specifically in the course that looks into the management and communication of disability-related information

### Themes generated in cross-case analysis

- Community-based interventions require contextually relevant tools.
- Identifying a contextually relevant tool requires consideration of a range of influences such as socio-economic, political, geographical, historical, structural, linguistic, and cultural (among others).
- For researchers to develop tools for equitable service delivery, they need a level of political consciousness.
- Contextual needs are identified through engagement with end-users.
- Collaborative research-clinical partnerships and relationships are necessary for developing tools.
- Scientific pilot and feasibility studies for tool development are necessary.
- Qualitative and quantitative methods are complementary in study design.
- Tools developed can be used when they are refined and validated.

A discussion of these themes is highlighted as lessons learned.

## Discussion: lessons learned

### Lesson 1: developing new tools and protocols, motivated by the need for contextually relevant practices, is essential

There were several dimensions of context that were addressed across the case studies. It was imperative that the socio-political and economic context was understood as a basis for developing tools that could potentially advance equitable practices. The politically conscious choice to prioritize populations who are unserved/underserved was necessary [5]. In case study 1, the population of school children in the public sector who struggled to receive effective primary-level care in a democratic South Africa was identified as a significant concern. Similarly, case study 2 targeted school children in public schools with minimal access to services who are subject to bullying because they stutter. Case study 3 identified persons with disabilities who require home-based care as an invisible population in low-income communities due to their severe impairments and disabling environments.

The case studies were sensitive to various contextual factors, including time and resource constraints resource of their rehabilitation service partners (teacher, nurses, and community rehabilitation workers). Nurses and community rehabilitation workers are required to operate within public settings where the health system is severely resourced-constrained impacting on how they practice in their everyday environments. Case study 1 focused on the nurses, addressing their need for a protocol that was effective and affordable and one that was applicable so that it could be administered rapidly to a large number of children. Similarly, community rehabilitation workers had significant time and resource constraints as they had to provide services to several persons with disabilities in the community and therefore needed a tool which could efficiently gather information and monitor how the intervention was proceeding. Likewise, teachers are part of public schooling which have timetables and large classes and require tools that can be integrated into their lessons.

Case studies 2 and 3 illuminate how the tools were developed to be responsive to the cultural and linguistic context. The CCR was revised until the content was considered culturally relevant by identifying concepts familiar to learners through cognitive debriefing. Furthermore, the stories used in the intervention as well as the outcome measure were revised until it was at a literacy level that was suited to learners who were learning English as a second language. Case study 3 invested in making the tool linguistically accessible through translating tools from English into IsiXhosa and Afrikaans. The translated tool created an opportunity to generate focused conversations thereby encouraging persons with disabilities to participate actively in defining their health needs, how they are met/unmet, and their priorities.

Contextually relevant tools therefore should be responsive to the multi-faceted nature of contexts by considering socio-political, economic, structural, cultural, and linguistic realities, among others.

### Lesson 2: partnerships and collaboration with end-users are critical for success

The learning in these case studies was that partnerships should be initiated at the point of developing tools and maintained throughout, rather than only at the implementation phase. In these case studies, the partners varied and included nurses, teachers, learners, and community rehabilitation workers. This collaborative approach to developing tools signals a shift away from the researcher-as-expert approach, where researchers develop tools based on their expert knowledge, to a more participatory approach which valued end-users as collaborative partners in co-producing knowledge. In the process, partners exercised their agency in identifying needs and reflecting critically on how their needs were being met. Their participation lays the groundwork for increasing the uptake of the tool in their practice settings.

### Lesson 3: a critical, scientific process is essential in developing new tools

The case studies show how qualitative and quantitative methods complemented each other in the development of tools and protocols and the assessment of feasibility. The value added was that knowledge was generated using systematic and rigorous methods in studies that were purposefully designed. In particular, the importance of a critical research paradigm [30] which acknowledges that knowledge is contextualized, contestable, power-laden, and driven by a need for social change was found to be valuable. This lesson is illustrated by drawing on two research methods.

#### Systematic literature review

In case studies 1 and 3, a systematic literature review contributed to the conceptual basis to draft the resource tool or protocol. For example, in case studies 1 and 3, the literature review appraised international literature related to disability, health, and hearing screening protocols. The literature was appraised to establish the applicability, strengths, and shortcomings, relative to the needs of the context in South Africa. The review illustrated the strengths and value of previously developed tools while also highlighting that it was necessary to develop measures that were fit for purpose for the South African context. The critical appraisal of established knowledge and its applicability was essential to assess the extent to which such knowledge could be meaningfully applied to the local context.

#### Expert panel: whose expert (ise) matters

To develop a contextually relevant resource, case study 3 showed that it was essential to identify the expertise in the field. An expert was defined as someone with relevant knowledge in an area of interest such as disability, rehabilitation, community-based rehabilitation, or the development of resource tools. Knowledge could be gained through practical experience/technical experience or through a professional qualification. While traditionally science-valued professionals and researchers, the expertise here was drawn by collectively engaging persons with disabilities, community rehabilitation workers, rehabilitation therapists, and academics.

The inclusion of people with disabilities was essential in case study 3. This is in alignment with the United Nations Convention on Rights of Persons with Disabilities UNCRPD [31], specifically article 4 and article 19. Article 4 of the UNCRPD seeks to ensure and promote “full realization of all human rights and fundamental freedoms for all persons with disabilities without discrimination of any kind on the basis of disability.” Article 19 states that community services should be responsive to the needs of persons with disabilities. The inclusion of an expert panel strengthened the validity of the resource tool and impacted positively on the development and refining of the tool [32, 33]. The experts shaped the essence of the tool, identified the content domains of the tool, and evaluated the strengths and shortcomings of the tool. The experts contributed to making the tool more suitable for the end-user. Methodologically, this approach contributed to the validity and robustness of the resource tool. Similarly, in case study 1 the expertise by experience was valued in including nurses in all stages of the research process. This inclusive approach to expertise resonates with the intention of Re-Aim framework which favors a scientifically based implementation approach that is contextualized and localized through the participation of key stakeholders [16].

### Lesson 4: pilot and feasibility studies are a critical part of developing tools and assessing feasibility

In all the case studies there was a pilot or feasibility study to review various aspects of feasibility. The researchers learned that while a tool may be developed scientifically, the feasibility of *implementation* must also be assessed with the participation of the end-user, e.g., nurses, teachers, or community rehabilitation workers. For example, with the hearing screening protocol in case study 1, the researchers learned that the tool met the needs of nurses and was practically implementable. However, researchers also learned that there were limitations in the procedural aspects of implementation which impacted negatively on the sensitivity of the protocol. Similarly, in case study 2, the pilot study [20] informed the design of an RCT through assessing the potential treatment effect of the CCR intervention as well as the feasibility of recruiting and retaining participants. The study concluded that an RCT was feasible subject to strengthening several procedural aspects of the process.

The pilot study of RHIT aimed at assessing the applicability of the tool by community rehabilitation workers with the population of persons with disabilities. The findings of the study helped the researcher and community rehabilitation workers to understand that the tool was part of a dynamic interactive process with persons with disabilities rather than a once-off information-gathering tool [27]. In contexts where tools are in the early stages of development, pilot and feasibility studies have been invaluable. Typically, they are smaller-scale studies that are more cost-effective and manageable and yield findings that address early-stage challenges, thus contributing to developing sustainable interventions.

### Lesson 5: the goal is to develop practical, usable tools and protocols

Each of the case studies demonstrated that it was feasible to develop tools that could be implemented practically. This goal has been achieved through relatively small-scale studies that show that systematic, scientific tool development is practical and possible — even within resource-constrained environments. The participatory approach provided an avenue to strengthen the relationships between researchers, service providers, patients, and end-users to work collaboratively to address the challenge of equitable health service delivery. While integrating tools into service delivery is a longer-term process that requires the interaction between policymakers, service users and providers, and researchers, these studies highlight that researchers have a key role to play in strengthening contextualized service delivery.

## Conclusion

The development of tools is a critical part of strengthening public health systems, particularly in the Global South. The case studies illustrate that it is both practical and possible to use scientific approaches to advance the development of tools and assess the feasibility of implementation through pilot and feasibility studies. The participatory approach to co-producing tools contributed to developing tools that were more responsive to contextual needs. Community-based rehabilitation service delivery is a growing critical international need and the scientific development of contextually relevant tools will contribute to addressing this service delivery challenge.

## Data Availability

All raw data is available upon request.
